# Pattern Unifies Autism

**DOI:** 10.3389/fpsyt.2021.621659

**Published:** 2021-02-12

**Authors:** Bernard Crespi

**Affiliations:** Department of Biological Sciences, Simon Fraser University, Burnaby, BC, Canada

**Keywords:** autism, repetitive behavior, systemizing, social cognition, pattern, psychosis

## Abstract

Autism is a highly heterogeneous condition, genetically and phenotypically. This diversity of causation and presentation has impeded its definition, recognition, assessment, and treatment. Current diagnostic criteria for autism involve two domains, restricted interests and repetitive behavior (RRBs) and social deficits, whose relationship remains unclear. I suggest that the large suite of traits associated with autism can be usefully conceptualized under the single rubric of “pattern,” a term that connects autism with basic brain and cognitive functions and structures its phenotypes within a single theoretical framework. Autism thus involves increases and enhancements to pattern perception, pattern recognition, pattern maintenance, pattern generation, pattern processing, and pattern seeking. RRBs result from increased and imbalanced pattern-related perception and cognition, and social alterations result in part from the usual lack of clear pattern in social interactions, combined with the interference of RRBs with social development. This framework has strong implications for assessment of social and non-social autism-related traits, personalized therapy, and priorities for research.

## Introduction

The development of assessment tools and therapies for autism requires increased understanding of its nature and causes, as a disorder and in each affected individual. Kanner (1) considered autism as a syndrome, that can be defined as a constellation of phenotypic traits and differences from typical individuals, that show some tendency to be found together within individuals, or that when found together cause psychological differences or problems. Each individual diagnosed with autism thus exhibits some subset of its many associated traits, each to some degree.

The constellation of autistic traits is divided by DSM5 into two domains, (a) social cognition, communication and behavior and (b) restricted interests and repetitive behaviors (RRBs). RRBs include stereotyped or repetitive actions, ritualized behavior, extreme adherence to routines, insistence on sameness, restricted interests, and alterations in reactivity to, and interest in, sensory stimuli. Under the DSM5 rubric, diagnoses of autism are categorical, and continuous variation in its constituent pheotypes is accounted for using estimates of severity. However, the relative roles of categorical and continuous variation in characterizing autism remain matters of intense inquiry (2–4).

The two domains of sociality and RRBs are weakly to moderately associated with one another (5, 6), and the causes of their connections remain unclear. Links between RRBs and sociality may thus involve common causes of both, one causing the other, reciprocal causation, or little to no causation at all. Developing and testing theory for the relationship of RRBs with sociality is important for understanding autism. Such theory must, moreover, be developed in the context of what adaptive phenotypes have become altered in autism to become problematic (7). The adaptive significance of social behavior and communication are evident, given that humans exhibit large, diverse brain regions subserving social cognition and tend to be highly socially interactive. By contrast, determining what adaptive traits have changed to generate RRBs, which include a tremendous diversity of phenotypes from self-stimulation, to stereotypy, to narrow interests in parts of objects or STEM disciplines and to savant skills, is more challenging. Is there any conceptual umbrella that can help to unify the diverse non-social, as well as social, phenotypes of autism, in the context of alterations to adaptive cognitive phenotypes?

At its most basic level, the function of the brain is to perceive and recognize patterns, transform them into information held in neuronal systems, process the information, and, when motivated to do so, act upon it in coherent ways (8, 9). Pattern perception, recognition, maintenance, processing, seeking, and generation thus represent the simplest and most general of neurological and cognitive functions, which in humans have evolved extreme complexity via expansion and specialization of neural subsystems.

In this short Perspective article, I propose, evaluate and apply the hypothesis that the dimensions of the concept of pattern can unify diverse autism phenotypes, across developmental, neurological, and psychological levels of analysis. This article thus presents the core of a unified framework for understanding autism in this way, set in the context of relevant previous theory.

I first describe what is meant here by pattern, and how this concept relates to previous theory for autism. Second, I explain a set of seven major dimensions of autism that can be conceptualized in terms of pattern and non-pattern, and provide evidence for the meaningfulness of each. Finally, I discuss implications for assessment of social and non-social cognition in autism, as well as for therapies and research.

## Pattern

Pattern is defined here as spatially or temporally repeated configuration, with recurring, ordered, or otherwise predictable characteristics, and discernable interrelationships of components. This conceptualization of pattern is closely allied to the presence of information, which can be considered from a variety of perspectives including sensory input, entropy, and predictability. Pattern connects especially directly with intelligence, which centrally involves the generation and inference of informational patterns. In this context, convergent evidence from previous work (7) indicates that autism can be characterized as a “disorder of high intelligence,” whereby it involves especially-high levels of the genetic, neurological and psychological components of intelligence, but also imbalances between its components, that mediate the development and expression of autistic phenotypes. Pattern is more general than intelligence, because it encompasses a broader array of perceptual, cognitive, and behavioral phenomena.

The concept of pattern is related to the concept of systemizing as developed by Baron-Cohen (10). Systemizing has been defined as the drive to observe, analyze or construct rule-based systems that function in an “if-then” manner, and it is clearly elevated in individuals with autism (10). As such, systemizing naturally encompasses aspects of pattern seeking, pattern processing, and pattern recognition, but not pattern perception, pattern maintenance, or pattern generation. It thus does not, in a clear or direct way, help to explicate the presence and forms of many important autism-related traits, including stereotypies, insistence on sameness, self-stimulation, lining up of objects, highly-fixated interests, or exceptional memory, realistic art or musical skills in savants. Given its “if-then,” rule-based structure, systemizing may, most generally, be considered as one component of high, human-evolved intelligence that is elevated in autism, leading to a cognitive imbalance characteristic of this condition.

### Pattern Perception

Pattern perception is based upon sensory perception. Sensory perception, measured as sensory reactivity or acuity, is often increased in autism, across different modalities; however, reactivity can also be decreased, leading to reduced stimulus responsiveness (11, 12). Several influential theories of autism, “enhanced perceptual function” theory (13) and the “intense world” theory (14), explain how increases in perceptual functions in autism can lead to some of its central phenotypes. As described in accounts from persons with autism (15), increased sensory sensitivity and acuity commonly result in sensory overload: too much incoming unpatterned and unfiltered information. Sensory overload is dealt with in several ways by individuals with autism: (1) reducing perception of sensory input as much as possible, resulting in sensory “hypo-reactivity” from an observer's perspective, or (2) self-stimulation or other simple repetitive behavior, or insistence on sameness, [e.g., (16, 17)] to calm the autonomic system by self-generating regularity; or (3) increases in focused attention on particular patterned stimuli, which leads to blocking out of other sensory inputs [e.g., (18, 19)]. Liss et al. (20) reported, for example, that about 40% of a large sample of autistic subjects showed a clear profile of high sensory sensitivity coupled with highly over-focused attention. Taken together, these findings can help to explain the apparently-paradoxical observations of especially high as well as low sensory sensitivities in autism, in the context of a pattern-based perspective.

Given that one of the primary functions of the brain is to extract information from sensory stimuli (8), enhanced sensory acuity, and increased attention to detail, may also increase the potential to obtain information from the world regarding patterns and thus may facilitate systemizing as well as high intelligence (7, 21). Sensory sensitivity, however, shows no evidence of association with systemizing among children with or without autism, but instead demonstreates a negative correlation with empathizing (22), suggesting that it may interfere with the acquisition of social abilities. By contrast, high sensory acuity has been positively associated with measures of intelligence across many studies tracing back to Galton and Spearman [reviewed in (7)]. The reasons for these associations remain unclear, but they strongly support the hypothesis that autism encompasses correlates, and imbalances, of high intelligence and highly-patterned perception and cognition.

### Pattern Recognition

Pattern recognition involves finding a particular visual-spatial pattern in a broader scene (as in the embedded figures test, EFT), and inferring what pattern comes next in a series of systematically changing visual forms (as in Raven's Progressive Matrices, RPM). Individuals with autism tend to show absolutely or relatively enhanced abilities in both of these tests, compared with control individuals, as well as in other tests and metrics of pattern recognition such as visual search [e.g., (23); reviewed in (7)]. Performance on both the EFT and RPM is positively correlated with general intelligence, and indeed the RPM serves as a metric of fluid intelligence (ability to think logically, identify patterns, and solve problems independently of acquired knowledge), which is enhanced in autism relative to crystallized intelligence (ability to solve problems using acquired knowledge) (24, 25). The recognition of repeating patterns in stimuli has also been proposed as a primary basis for forms of talent and savantism in autism, such as calendar calculating, mathematics and other specialized skills (21, 26).

Might enhanced pattern recognition be causally linked to RRBs, or sociality, in autism? Better performance on the EFT has traditionally been considered to reflect “weak central coherence,” which can be construed as a local pattern processing bias and relative inability to see the “big picture” (27). It can also be seen to indicate high sensory acuity combined with stronger attentional focus and working memory, both of which are associated with high intelligence (7). In principle, local processing biases and sharper attentional focus can synergistically contribute to intense, narrow interests as found in some RRBs, because higher-level structures (“wholes”), which are not necessarily repeated, are not perceived (27, 28). As such, a focus on pattern can also help to explain increased local compared to global information processing in autism, as a cognitive imbalance with both benefits and costs in different psychological contexts.

### Pattern Maintenance

Insistence on sameness represents a uniquely autistic trait. This phenotype can be described in terms of the temporal maintenance of current patterns in the environment. Insistence on sameness and associated ritualistic behaviors are developmentally typical for children aged about 2–4 years [see (29)], but in autism they extend into much later ages. The intensity and developmental persistence of insistence on sameness appear to be mediated by some combination of high sensory sensitivities and high attention to details in the environment, as evidence by high reactivity on ERP oddball tasks of novelty detection [e.g., (30)], higher mismatch negativity (31), and reduced levels of inattentional blindness (32). Individuals with autism also tend to score lower than controls on the temperament trait of novelty-seeking (33). Finally, pattern maintenance in autism may also encompass overselective attention (18, 34), which serves to actively maintain sameness. Pattern maintenance can thus describe a range of autism-related traits, especially ones that are difficult to explain from other theoretical perspectives.

### Pattern Generation

Pattern generation in its overt form represents repetitive, stereotypic behaviors and self stimulation. These behaviors are postulated to help individuals with autism to cope with strong and uncontrolled, unpredictable sensory inputs, and accompanying stress, by producing a pattern that can mask or override them, or calm the autonomic nervous system [e.g., (17)], as noted above. Pattern generation may also be covert and cognitive, whereby individuals with autism engage in repetitive thought patterns, reflected for example in the recent finding of higher organizational activity in the default mode, the brain system that underlies stimulus-independent thought, in autistic individuals (35).

The nature of pattern generation is notably associated with overall intelligence in individuals with autism, in that stereotyped behaviors such as motor mannerisms are found more commonly in autistic individuals of lower intelligence, while more-complex ritualized behaviors are more characteristic of autistic individuals with higher intelligence (36). This variation appears to reflect the complexity of neurologically-based patterns that can be sustained under different levels of general intelligence, and different levels of its imbalance in components, such as sensory acuity in relation to capacity for logic and self-regulated executive functions.

### Pattern Seeking

Pattern seeking involves cognitive drive to find and confirm patterns in the external environment. This phenomenon is closely allied to systemizing, though it also involves higher explanatory drive in non-social problem-solving in autism (37), and higher, albeit imbalanced, intelligence (7), given that systemizing is not positively associated with intelligence [e.g., (38)]. Enhanced pattern seeking in autism is also manifest in, for example: (a) pursuits in highly-organized collecting and related aspects of systemizing and (b) the associations of the autism spectrum with interests and skills in STEM disciplines [e.g., (10, 39)]. Pattern seeking and autism in the context of STEM disciplines are also closely related to high intelligence, and imbalances in intelligence, given the especially high intelligence reported among individuals in these disciplines [see (7)].

### Pattern Processing

Pattern processing is logical, organized transformation of patterned information that involves problem solving, decoding, or other mental manipulation of cause-effect relationships. Evidence for enhancement of this cognitive domain in autism includes (a) hyperlexic decoding of written words and other examples of veridical mapping (40), (b) a more deliberative than intuitive cognitive style, by the cognitive reflections test or jumping to conclusions protocols (41, 42), (c) increased logical consistency (43, 44), (d) less influence from prior beliefs and experiences in conditional reasoning tasks (45), (e) better syllogistic reasoning skills (46), and (f) enhanced performance in the Iowa Gambling Task compared to controls (47). Pattern processing also represents a core component of intelligence and the task-positive network, that mediates between information input and decision-making (7).

The six non-social dimensions of pattern in autism described above are related to one another in their links with neural-system models of intelligence, especially the Parietal-Frontal Integration Theory (P-FIT) [see (7)]. Thus, pattern perception and generation are associated with Sensory Processing in the P-FIT model, pattern recognition and maintenance are associated with Abstraction and Generalization of sensory information in the model, and pattern seeking and processing are associated with Hypothesis Testing and Decision Making. These connections provide a hypothesis for an explicit neural basis to pattern in autism, grounded in the sequential processes by which the human brain takes in, processes, and acts upon information.

### Non-pattern Avoidance

The considerations described above show that virtually all non-social autism-related traits can be conceptualized in terms of relative increases and imbalances in pattern perception, recognition, maintenance, generation, seeking, and processing. A simple corollary of these observation is that non-pattern should be avoided. Social interaction and cognition exhibit low levels of predictable pattern because they result, in large part, from idiosyncracies in the minds of others. These are intrinsically not computable or generalizable except in weakly-probablistic ways, being dependent on emotions that may not even be perceived by the actors themselves. These considerations do not deny the existence of social patterns, but note that they involve brain systems that are temporally incompatible with non-social ones (48) as well as being less predictable.

How, then, might RRBs and patterned cognition be related to sociality? A suite of evidence indicates that social skills, empathizing, or verbal skills are inversely related to (a) sensory sensitivity (22), (b) visual spatial abilities (49), (c) systemizing (50), (d) EFT scores (51–53), (e) RPM scores (54), (f) visual search skills (55, 56), and (g) mathematical ability (57). These findings suggest that pattern-related cognition, as a phenotype that is broadly and deeply increased or enhanced in autism, may pre-empt, preclude or trade off with phenotypes that are absent or reduced: social skills and interests. For assessment and therapy, the important questions to address involves if and how this occurs, and what to do about it.

## Discussion

**Figure 1:**
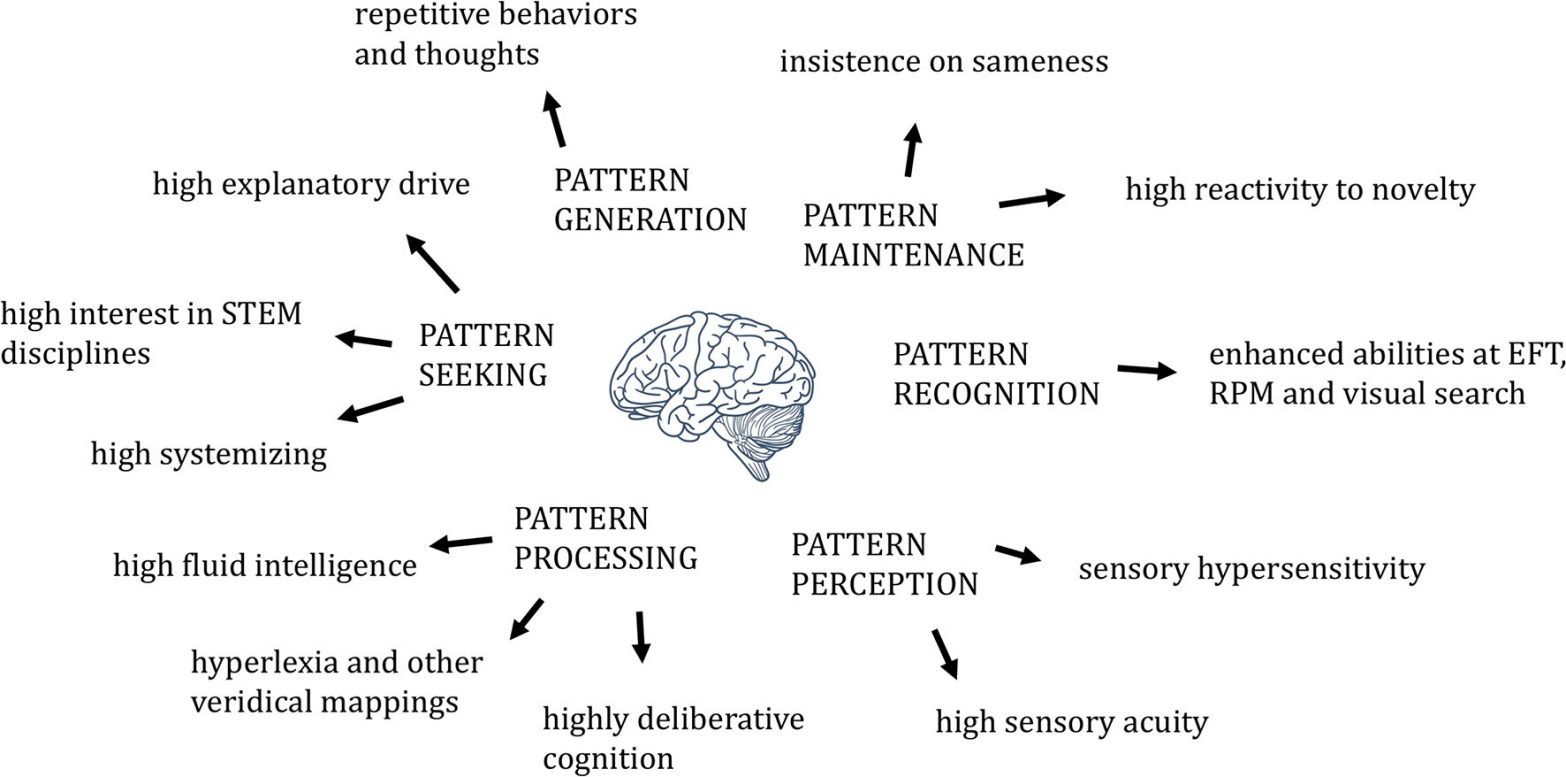
The six non-social dimensions of pattern, in relation to relevant autism-associated phenotypes.

The human brain is an engine for pattern perception, recognition, maintenance, generation, seeking, and processing. Brains of individuals with autism show evidence of increases or enhancements in each of these processes (Figure 1), though only in some subset of the six for any given individual with autistic traits. Such imbalances may, like imbalances in components of intelligence more generally (7), favor the development of what we call autism.

The usefulness of the pattern-centric framework for autism described in this Perspective is conceptual unity, which provides guidance toward ways to better study its causes, ways to better assess individuals with autistic traits, and ways to develop better treatments. A focus on the domains of pattern also connects more directly with specific neurological structures and functions than do other conceptual models [e.g., (7)], and can thus help to encompass the wide heterogeneity among autistic individuals. For example, convergent evidence suggests that autism is associated with increased hippocampus-mediated pattern separation, which may help to explain aspects of sensory sensitivity and pattern recognition ability, with direct impacts on regulation of social-emotional behavior in some autistic individuals (58–60).

The main upshot of these inferences is that for individuals with autism, social cognition cannot be thoroughly or properly assessed without consideration of the structure of non-social cognition. Thus, far, such assessment is mainly limited to analysis of sensory symptoms, stereotypies, and systemizing, without a coherent analytic framework for linking them to other traits, to each other, or to social and typical cognition. In principle, the non-social, pattern-related phenotypes of autism should connect with the social ones, through pre-emption, biasing, tradeoffs, and other forms of association.

Under the framework described here, and given some understanding of how the domains of pattern are altered in individuals with autism, therapies can develop in one of two main ways: (a) patterning of sociality and (b) socializing of patterns.

Patterning of sociality involves attempts to make social interactions more understandable and predictable, by treating other individuals as pattern-driven beings. This approach can be called “systemizing” of sociality (61), whereby animate beings are “deanimated” into causal, if-then patterns as best possible. This is an intellectual, reverse-engineering process, and is thus only likely to work well in individuals with autism of relatively high general intelligence.

Socializing of patterns involves working from inanimate to animate, via the incorporation of metarepresentation, imagination and novelty into the highly-patterned cognition elements of individuals with autism [e.g., (62)]. “Things” are thus transformed into forms and components of “people.” This approach involves the overlay of imaginative non-pattern onto patterned elements of cognition and behavior, to move them in the direction of typicality and toward completion of typical development (29). Given the extensive evidence of opposite cognitive phenotypes in autism compared to psychosis and schizophrenia (63), this direction is also the direction of non-pathological positive schizotypy, which can provide guidance as to what specific traits to encourage, such as imagination, animation, novelty-seeking, and openness. The nature of such therapies should take account of the non-social, patterned cognitive system of each particular individual with autism, to understand how they fit with the domains of pattern described above.

Kanner (1) discussed non-social autistic traits in great detail, and intimated their links with extreme autistic aloneness. However, he never mentioned social deficits *per se*, which appear to be the main emphasis of autism research at present. A return to focus on pattern and non-social cognition in autism, in the context of their links to sociality, should lead to useful insights.

## Data Availability Statement

The original contributions presented in the study are included in the article/supplementary material, further inquiries can be directed to the corresponding author.

## Author Contributions

The author confirms being the sole contributor of this work and has approved it for publication.

## Conflict of Interest

The author declares that the research was conducted in the absence of any commercial or financial relationships that could be construed as a potential conflict of interest.
